# What is special about our own face? Commentary: Tuning of temporo-occipital activity by frontal oscillations during virtual mirror exposure causes erroneous self-recognition

**DOI:** 10.3389/fpsyg.2015.01551

**Published:** 2015-10-13

**Authors:** Maria L. Filippetti

**Affiliations:** Department of Psychology, Royal Holloway, University of LondonEgham, UK

**Keywords:** self-recognition, body perception, multisensory integration, self-updating, body awareness, face recognition

*‘What are you doing?’, my wife asked, seeing me linger, unusually in front of the mirror*.

‘Nothing’, I replied. ‘Just looking at myself, at my nose, here, inside this nostril. When I press it, I feel a little pain.’

My wife smiled and said: ‘I thought you were looking to see which way it tilts.’

*I wheeled around like a dog whose tail has been stepped on*.

‘Tilts? My nose?’

Pirandello (1990)

Observing one's face in the mirror provides an immediate strong sense of self. The ability to recognize one's face has been taken as an index of self-awareness and has been considered a fundamental aspect within the spectrum of selfhood (Gallup, 1970; Rochat, 2009). However, what is considered the most representative instance of personal identity is probably the less reliable representation of the self; the rather infrequent encounters we have with our own face are in fact mediated by a reflecting surface and likely distorted by a variety of expectations shaped by ourselves and others (Rochat and Zahavi, 2011). How do we balance this information in order to maintain a contentment and stable mental representation of our own identity? A new study by Serino et al. (2015) provides evidence of the neural mechanisms that mediate the balance between online multisensory cues and offline stored self-representation for the update of self-face recognition.

That self-face representation is plastic and susceptible to changes is not to be news. Recent studies (Tsakiris, 2008; Paladino et al., 2010; Sforza et al., 2010; Tajadura-Jiménez et al., 2012a,b) have applied the principles of multisensory integration of body-related signals (e.g., Botvinick and Cohen, 1998; Tsakiris and Haggard, 2005) to induce a sense of identification with a new face by creating the Enfacement Illusion (EI). In the EI, the sight of an unfamiliar face being touched on her cheek together with the participant's own face creates a “mirror-like” experience and elicits a change in self-face recognition. The perfect correspondence between what participants see and what they feel on their face updates the offline stored self-representation, albeit the face identity they see is a different one. As a result, the other person's face observed during the Enfacement becomes included in the mental representation of one's own face (Tsakiris, 2008). While Apps et al. (2015) used fMRI to explore the neural underpinnings of self-identification and self-updating, Serino et al. (2015) now provide further evidence of the role of online multisensory processing in self-face recognition. This research makes a relevant contribution to the state of art in two fundamental ways. First, by employing visuo-motor information (rather than the classic visuo-tactile feedback used in the EI), the authors investigate the role of the most commonly experienced multisensory information when facing one's own mirror reflection. By adopting a virtual-mirror setup, Serino et al. (2015) show that the visuo-motor correspondence between self and avatar's movements elicits a change in self-face recognition. In line with the EI, after being exposed to visuo-motor synchrony, participants report the other face as resembling more one's own face. Second, this study provides crucial evidence of the mediating effect of the online multisensory mechanism on the offline self-representation by showing that sensory-motor and inferotemporal-occipital areas of the cortex are both involved in self-face recognition (Serino et al., 2015).

These converging neuroimaging findings corroborate the existing evidence that in adults the mental representation of one's face can be altered by multisensory processing, but what happens when this offline self-representation is not yet in place? Different psychological accounts have historically tried to explain the developmental markers of self-face recognition. The reference paradigm used in most of these studies is the “mirror-test,” which consists in placing a red spot in the child's forehead or nose and test whether, when faced with a mirror, the child notices the spot (Gallup, 1970; Amsterdam, 1972). Children's successful attempt to reach for the spot is taken as evidence of self-recognition. Despite the extensive research done using Gallup's mirror-test for the investigation of self-recognition in chimpanzees (Gallup, 1970), dolphins (Parker et al., 1994), elephants (Plotnik et al., 2006), and human infants (e.g., Amsterdam, 1972; Bertenthal and Fischer, 1978; Povinelli et al., 1996), the paradigm has been criticized because of the high variability of performance across ages (Rochat, 2007, 2009) and cultures (Broesch et al., 2010), and its strict criteria (Bahrick et al., 1996). With regard to the latter point, the idea that self-consciousness is a unitary phenomenon, which can be measured through a “yes/no” task, has been rejected by psychologists and philosophers. In fact, in order to recognize themselves, infants must rely on the exploration of the perfect correspondence of sensory information and match the seen featural cues of the visual appearance to the self. The mirror-test disregards the relationship between these two complex components. While recent evidence suggests that some predisposition to multisensory body-related cues is present soon after birth (Filippetti et al., 2013, 2015), the presence of an in-built representation of self-appearance is unlikely, and infants encountering a mirror must match the motor information with the reflected visual output and eventually link the featural cues of the reflection with themselves.

Serino and colleagues' findings (2015) provide a fundamental starting point for research on the developmental processes involved in self-recognition and self-other differentiation. On one hand, their evidence of the presence of neural mechanisms responsible of the mediation between online and offline self-recognition in adults can pave the way for future investigation on the brain processes involved in the developing self in infancy and childhood. Additionally, evidence from neuroimaging can disentangle the respective roles of physical (e.g., self-reflection) and social (e.g., imitative interaction) mirrors, by exploring how the infant brain responds to variations in multisensory correspondences and visual appearance. At present, the developmental origins of self/other differentiation are still unknown and one of the pressing questions is whether the self emerges as a consequence of repetitive mirroring interactions with others, rather than as a-priori fundament of human beings (Prinz, 2013). While significant information about the self can be exploited from interactions with others, I posit that self-recognition is the result of both self-exploration *and* exchanges with others. Through a process of balance between multisensory processing and experience with one's facial appearance, sensory-motor and face-related areas of the cortex mediate this interaction. The findings from Serino et al. (2015) provide the starting point of investigation for future developmental research in this direction.

## Conflict of interest statement

The author declares that the research was conducted in the absence of any commercial or financial relationships that could be construed as a potential conflict of interest.
